# Effect of chronic heat stress on duodenal epithelial barrier integrity in low- and high-water-efficient broiler chickens

**DOI:** 10.3389/fphys.2025.1704737

**Published:** 2025-11-13

**Authors:** Lulu Liu, Elizabeth S. Greene, Brooklee Roach, Sara Orlowski, Sami Dridi

**Affiliations:** Center of Excellence for Poultry Science, Division of Agriculture, University of Arkansas, Fayetteville, AR, United States

**Keywords:** heat stress, intestinal barrier integrity, tight junction, water efficiency, gene expression

## Abstract

Heat stress (HS) has long posed a significant challenge to the poultry industry due to its adverse effects, such as depressed feed intake, decreased growth performance, and increased water consumption. Water efficiency (WE, conversion of water intake into body weight gain), although often neglected, is a key economic and production trait that is significantly affected by HS. Recently, we selected two broiler lines for high WE (HWE) and low WE (LWE) and showed a differential hypothalamic expression of genes involved in water homeostasis regulation. As the gut also plays a significant role in water absorption, the present study aimed to determine the effect of chronic HS on duodenal barrier integrity in LWE and HWE broilers. Male HWE and LWE chicks (240 chicks/line) were individually wing-banded for line identification, weighed, and placed in 12 controlled environmental chambers (2 pens/chambers). On day 29, birds were subjected to thermoneutral conditions (TN, 25 °C) or cyclic HS conditions (HS, 36 °C for 9 h/day from 9:00 a.m. to 6:00 p.m.) (120 birds/line/environment) for 3 weeks. On day 49, duodenal tissues were collected for histological, biochemical, and molecular analyses. Hematoxylin and eosin (H&E) staining revealed that HS significantly reduced villus height in the duodenum. Further analysis using qPCR showed that the mRNA expressions of intestinal barrier integrity-related genes, including claudins (CLDN1, 4, 5, 8, 16, and 22), PALS1-associated tight junction protein (*PATJ*), gap junction alpha 1 and 3 (*GJA1/3*), cadherin 2 (*CDH2*), and catenin alpha 2 (*CTNNA2*), were significantly upregulated by HS, and this effect was more pronounced in the HWE line than in its LWE counterpart. The findings of this study indicate that HS induces duodenal morphometric alterations. Based on the reduced serum fluorescein isothiocyanate-dextran (FITC-D) levels previously reported in the HWE line, the increased abundances of *CLDN*, *PATJ*, *GJA1*, *CDH2*, and *CTNNA2* mRNAs in the HWE line suggest an enhancement of its duodenal barrier integrity for better nutrient and water absorption and, consequently, better growth efficiency.

## 1 Introduction

Due to its nutritious characteristics (high protein content, low fat levels, and richness in vitamins) (Marcincak et al., 2023), poultry meat is highly regarded and widely consumed globally. To meet requirements of an ever-increasing human population of approximately 10 billion by 2050, it is required that global poultry meat production reach 181 million tons (Kikusato and Toyomizu, 2023) This high demand for high-quality animal proteins also necessitates rapid growth and increased broiler production efficiency, which will be very challenging because of many obstacles, particularly global warming (Oke et al., 2024). Heat stress (HS) is one of the most significant economic, production, and welfare burdens that adversely impact the poultry industry. According to a study on the world agricultural economy, HS results in $2.36 billion in economic losses annually to the U.S. poultry industry (Abbas et al., 2025) due to depressed feed intake, diminished production performance, suppressed immune function, heightened disease outbreaks, increased water consumption, and elevated mortality rates (Nawab et al., 2018; Liu et al., 2020). Depending on its duration, intensity, and severity, the adverse effects of HS can range from discomfort to organ damage and, in grave cases, to death.

Among the organs that are sensitive to HS is the gut. HS increases peripheral blood circulation and reduces blood flow in the intestinal epithelium, leading to hypoxia. This hypoxic condition compromises intestinal mucosal damage by disrupting tight junctions (TJs) and increasing intestinal paracellular permeability (Lambert, 2009). Furthermore, HS impairs various physiological functions of the gastrointestinal tract, such as digestive enzyme activity, nutrient transport, and intestinal development (Kikusato and Toyomizu, 2023), leading to increased inflammation (Varasteh et al., 2015; Wang et al., 2022) and leaky gut syndrome (Ncho, 2025).

Under homeostatic conditions, the intestinal epithelial cells form physical and biochemical barriers to prevent pathogens, toxins, and allergens from entering the intestinal lumen (Alhota et al., 2021). This intestinal epithelial barrier is formed by several complex components, including the adhesive mucus gel layer, immunoglobulin A, antibacterial peptides, and the apical junctional complex (AJC). The AJC is composed of TJs, adherens junctions (AJs), and desmosomes, together with gap junctions, which reside below AJC, conferring intestinal structural integrity (Suzuki, 2020; Garcia-Hernandez et al., 2017; Kuo et al., 2022; Gierynska et al., 2022).

The gut, called the second brain, plays a pivotal role in regulating water consumption, and both, as described above, are affected by HS. Two chicken lines were divergently selected for high water efficiency (HWE) and low water efficiency (LWE) (Aloui et al., 2024), with the HWE line being more thermo-resistant, exhibiting lower leaky gut syndrome under HS conditions (Greene et al., 2025), and differential hypothalamic expressions of genes involved in thirst and water homeostasis regulation (Aloui et al., 2024). Recently, we have also shown that HS affects ileal barrier integrity in a line-dependent manner (Greene et al., 2025). To deepen our understanding, the present study was undertaken to determine the effect of chronic HS on duodenal barrier integrity, which plays a crucial role in digestion and nutrient absorption, in HWE and LWE lines.

## 2 Materials and methods

### 2.1 Animal experiments and tissue collection

The LWE and HWE lines and the experimental design used in this study were previously described (Aloui et al., 2024). In brief, on day 1, male HWE and LWE chicks were randomly allocated in body weight-matched groups into 12 controlled environmental chambers (2 floor pens/chamber, 6 chambers/line, 20 birds/pen). On day 29, birds were exposed to two environmental conditions: thermoneutral (TN, 25 °C) or chronic cyclic HS (36 °C for 9 h/day) in a 2 × 2 factorial design. On day 49, the duodenum tissues (3–5 cm central portion of the duodenal loop) (n = 12/group) were rinsed twice in cold PBS 1X and snap-frozen in liquid nitrogen and stored at −80 °C for later use. Segments of the duodenum tissue were fixed in 4% paraformaldehyde for histological analysis. Animal care complied with the requirements of the Guide for the Care and Use of Laboratory Animals from the National Institutes of Health. All animal experiments were performed in accordance with the procedures approved by the University of Arkansas Animal Care and Use Committee (protocol # 23015).

### 2.2 RNA extraction, cDNA synthesis, and quantitative real-time PCR

Total RNA was extracted from duodenum tissues using TRIzol reagent (Thermo Fisher Scientific, Waltham, MA), according to the manufacturer’s instructions. RNA integrity and quality were assessed using 1% agarose gel electrophoresis, and RNA concentrations and purity were determined for each sample using a Take3 Microvolume Plate with a BioTek Synergy HT Multimode Microplate Reader (BioTek Instruments, Inc., Winooski, VT). Total RNAs (1 µg) were reverse-transcribed to cDNA using qScript cDNA SuperMix (Quanta Biosciences, Gaithersburg, MD) and subjected to quantitative real-time PCR with SYBR Green Master Mix on a 7500 Real-Time PCR System (Applied Biosystems, Waltham, MA) as previously described (Aloui et al., 2024). The qPCR reaction mixture consisted of 2.5 μL of cDNA, 5 μL of SYBR Green Master Mix (ABclonal, Woburn, MA), and 0.5 μL of each forward and reverse primer to make a final reaction mixture of 12.5 μL. Melting curve analysis was performed at the end of the amplification, following the dissociation protocol (Sequence Detection System) to exclude contamination with nonspecific PCR products. The PCR products were also confirmed through 2% agarose gel electrophoresis, which exhibited only one definite band of the predicted size, and by sequencing the amplified amplicons. There were no gel-detected bands for the negative controls where the RT products were omitted. The relative quantification of target gene expression was calculated using the 2^−△△CT^ method (Schmittgen and Livak, 2008), with the ribosomal *18S* gene as the housekeeping gene. The specific primer sequences used in this study were identical to those described in the previous study (Greene et al., 2025).

### 2.3 Western blot

Proteins were extracted from duodenum tissues using lysis buffer supplemented with protease and phosphatase inhibitors, as previously described (Greene et al., 2025). In brief, protein concentrations were determined using a Bradford Assay Kit (Bio-Rad, Hercules, CA), according to the manufacturer’s instructions. Equal amounts of protein (70 µg) were separated using 4%–12% Bis-Tris gels (Life Technologies, Carlsbad, CA) and transferred into PVDF membranes. The membranes were blocked at room temperature for 1 h with 5% non-fat milk in TBS-T and then incubated overnight at 4 °C with primary anti-CLDN4 antibody (1; 1,000, bs-2790R, Bioss, Woburn, MA) and anti-GAPDH (1:1,000, NB300-327, Novus Biologicals, Centennial, CO). After washing, the membranes were incubated with HRP-conjugated secondary antibodies (goat anti-rabbit IgG #7074, 1:5,000, Cell Signaling, Danvers, MA) for 1 h at room temperature. Protein bands were visualized using enhanced chemiluminescence (ECL) reagents (SuperSignal West Femto Maximum Sensitivity Substrate, Thermo Fisher Scientific, Waltham, MA) and captured using the FluorChem M MultiFluor System (ProteinSimple, Santa Clara, CA). Band intensities were quantified using AlphaView software (version 3.4.0.0, ProteinSimple, Santa Clara, CA).

### 2.4 Immunohistochemical and intestinal morphometry measurement

Duodenum segments were fixed in freshly prepared 4% paraformaldehyde for 24 h at 4 °C, dehydrated through a graded ethanol series, cleared in xylene, and embedded in paraffin. Paraffin blocks were sectioned at a thickness of 5 μm using a microtome and mounted on glass slides. Hematoxylin and eosin (H&E) (1%) staining was performed for duodenal morphology examination and measurement. Tissue sections were deparaffinized in xylene (3 × 15 min) and rehydrated through descending concentrations of ethanol (100%, 95%, 70%, and 50%, 3 min each). After rinsing in distilled water, tissue sections were stained with hematoxylin (VWR, Radnor, PA) for 20 min, briefly differentiated in 1% acid alcohol, and blued for 10 min. Tissue sections were then counterstained with eosin (VWR, Radnor, PA) for 20 min, dehydrated in ascending ethanol concentrations (95% and 100%, 3 min for each), cleared in xylene, and cover-slipped using mounting medium. Tissue morphometry was examined under a Nikon light microscope and using NIS-Elements software F (5.22.00, Nikon Instruments Inc., Melville, NY). The morphometric measurements on the duodenum included villus height (VH, from the top of the villi to the villus-crypt junction) and crypt depth (CD, from the base of the villi to the mucosa). All measurements were taken from 20 random villi and 20 random crypts from the duodenal segment of each bird and were expressed as the average villus height and crypt depth. The villi/crypt ratio was determined by dividing the villus height by the crypt depth (VH/CD) (Prakatur et al., 2019). ImageJ software was used to analyze all the measurements.

### 2.5 Statistical analysis

Data are shown as the mean ± SEM. Statistical analyses were conducted using GraphPad Prism version 10 for Windows (GraphPad Software, La Jolla, California, United States). For experiments involving line, environment, and their interaction, two-way ANOVA was performed, followed by Tukey’s honestly significant difference (HSD) multiple comparison test. If no significant interaction was detected, the main effects of the line or environment were evaluated separately using Student’s t-test. *p* < 0.05 was considered statistically significant.

## 3 Results

### 3.1 Morphometric analysis

**FIGURE 1:**
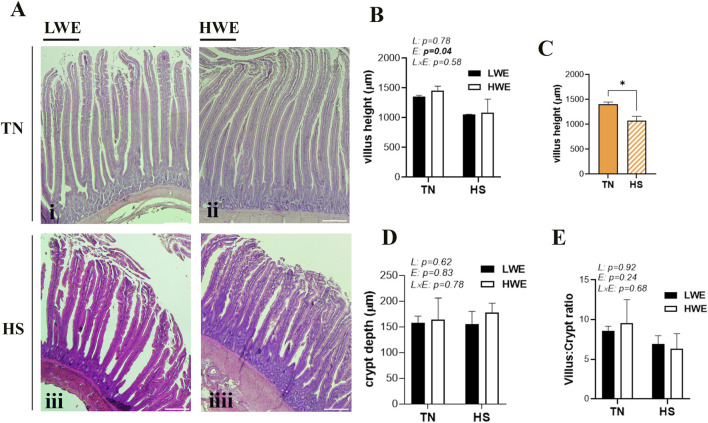
Duodenal histological morphology in LWE and HWE broiler chickens. **(A)** Representative H&E-stained sections of duodenum from LWE and HWE lines under TN and HS conditions. **(B,C)** Quantification of villus height (VH), **(D)** crypt depth (CD), and **(E)** the VH:CD ratio. Data are presented as the means ± SEM. Scale bar, 100 μm. * indicates a significant difference at *p* < 0.05. E, environment; HS, heat stress; HWE, high-water-efficient; L, line; L × E, line-by-environment interaction; LWE, low-water-efficient; TN, thermoneutral. A bold *p*-value indicates a significant difference.

Morphometric parameters of the duodenum from LWE and HWE lines reared under TN and HS conditions are shown in Figure 1. There was no significant line-by-environmental interaction effect for any of the measured parameters (Figures 1B,D,E). Heat stress significantly decreased the duodenal villus height compared to the TN condition (Figures 1B,C), but it did not affect crypt depth (Figure 1D) or the villus/crypt ratio (Figure 1E).

### 3.2 Effect of HS on the duodenal expression of tight junction proteins in HWE and LWE lines

**FIGURE 2:**
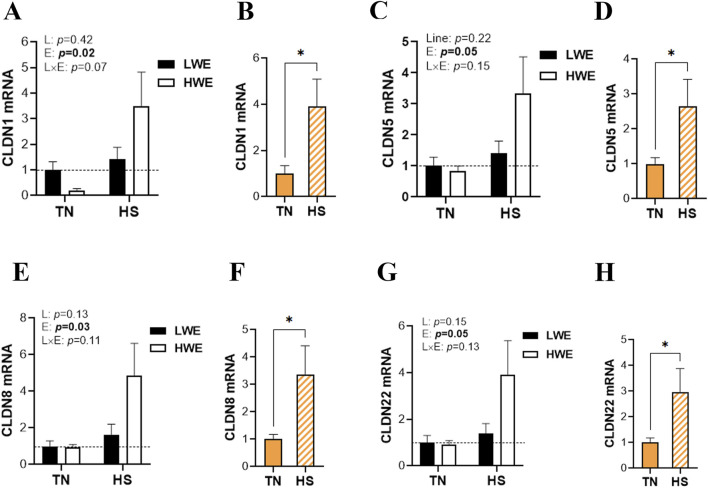
Effect of HS on the duodenal expression of barrier-forming claudins in HWE and LWE lines. Duodenal expressions of CLDN1 **(A,B)**, CLDN5 **(C,D)**, CLDN8 **(E,F)**, and CLDN22 **(G,H)** were determined using qPCR and 2^−ΔΔCt^, with 18S rRNA as a housekeeping gene and LWE-TN or TN as a calibrator. Data are presented as the means ± SEM (n = 6/line/environmental condition). * indicates a significant difference at *p* < 0.05. CLDN, claudin; E, environment; HS, heat stress; HWE, high-water-efficient; L, line; L × E, line-by-environment interaction; LWE, low-water-efficient; TN, thermoneutral. A bold *p*-value indicates a significant difference.

Heat stress exposure affects the duodenal expression of barrier-forming claudins. In particular, mRNA abundances of duodenal *CLDN1*, *CLDN5*, *CLDN8*, and *CLDN22* were significantly increased by HS compared to TN conditions (Figures 2A–H), and this induction was more pronounced in HWE than in their LWE counterparts (Figures 2A–H). The expressions of the *CLDN9*, *CLDN25*, and *CLDN34* genes remained unchanged between both lines under both environmental conditions (Table 1).

**TABLE 1:** Effect of HS on the duodenal expression of barrier integrity-related genes in HWE and LWE lines.

Environment (E)^a^	TN		HS		*p*-value		
Gene^b^/Line (L)^c^	LWE	HWE	LWE	HWE	L	E	L × E
CLDN9	1 ± 0.25	1.78 ± 0.56	1.01 ± 0.17	2.94 ± 1.13	0.06	0.39	0.39
CLDN25	1 ± 0.32	1.03 ± 0.26	0.90 ± 0.30	2.98 ± 1.11	0.11	0.16	0.12
CLDN34	1 ± 0.22	1.00 ± 0.21	1.10 ± 0.22	2.48 ± 0.80	0.14	0.09	0.14
CLDN19	1 ± 0.30	1.00 ± 0.22	1.06 ± 0.30	2.92 ± 0.98	0.11	0.09	0.11
CLDN23	1 ± 0.15	1.16 ± 0.05	1.20 ± 0.39	2.04 ± 0.58	0.20	0.16	0.38
CGN	1 ± 0.16	1.35 ± 0.34	1.37 ± 0.17	1.83 ± 0.39	0.17	0.14	0.84
OCLN	1 ± 0.20	1.06 ± 0.16	1.11 ± 0.19	1.69 ± 0.36	0.21	0.14	0.30
JAMA	1 ± 0.15	1.07 ± 0.15	0.85 ± 0.09	1.15 ± 0.16	0.21	0.82	0.42
GJC2	1 ± 0.28	1.34 ± 0.32	1.42 ± 0.40	2.36 ± 0.78	0.55	0.17	0.22
GJD2	1 ± 0.27	1.14 ± 0.26	1.22 ± 0.35	3.23 ± 1.13	0.11	0.09	0.16
CDH1	1 ± 0.33	0.96 ± 0.31	0.99 ± 0.30	2.25 ± 0.79	0.23	0.20	0.20
CTNNB1	1 ± 0.11	1.06 ± 0.10	1.18 ± 0.21	2.28 ± 0.63	0.12	0.06	0.16
AFDN	1 ± 0.25	0.66 ± 0.10	1.27 ± 0.36	2.00 ± 0.66	0.64	0.06	0.21
Nectin1	1 ± 0.28	0.90 ± 0.24	0.61 ± 0.04	1.07 ± 0.18	0.40	0.60	0.18
DSG2	1 ± 0.33	0.67 ± 0.24	0.96 ± 0.25	1.26 ± 0.27	0.95	0.32	0.26
DSG4	1 ± 0.37	0.76 ± 0.37	0.91 ± 0.28	1.36 ± 0.30	0.74	0.45	0.30

^a^ HS, heat stress; TN, thermoneutral.

^b^ AFDN, afadin; CDH, cadherin; CGN, cingulin; CLDN, claudin; CTNNA/B, catenin; DSG, desmoglein; GJC2, gap junction gamma 2; GJD2, gap junction delta 2; JAMA, junctional adhesion molecule alpha; OCLN, occludin.

^c^ HWE, high-water-efficient; LWE, low-water-efficient.

**FIGURE 3:**
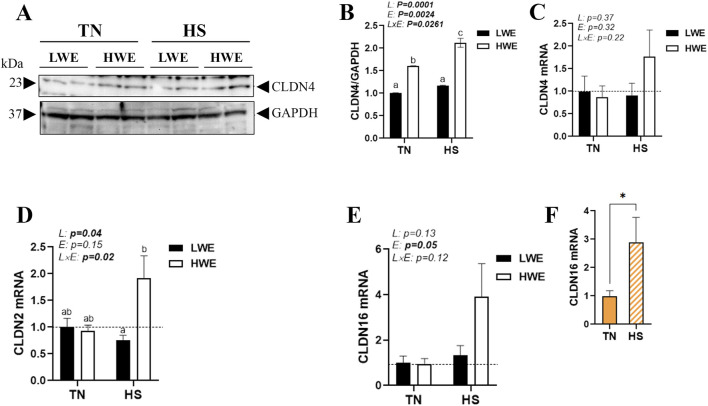
Effect of HS on the duodenal expression of pore-forming claudins in HWE and LWE lines. Protein levels of CLDN4 **(A,B)** were determined using Western blot. mRNA abundances of CLDN4 **(C)**, CLDN2 **(D)**, and CLDN16 **(E,F)** were measured using qPCR and 2^−ΔΔCt^, with 18S rRNA as a housekeeping gene and LWE-TN or TN as a calibrator. Data are presented as the means ± SEM (n = 6/line/environmental condition). Different superscript letters and * indicate significant differences at *p* < 0.05. CLDN, claudin; E, environment; HS, heat stress; HWE, high-water-efficient; L, line; L × E, line-by-environment interaction; LWE, low-water-efficient; TN, thermoneutral. A bold *p-*value indicates a significant difference.

The duodenal expression of pore-forming claudins was also affected in LWE and HWE broiler chickens. There were significant line-by-environment interaction effects for duodenal CLDN4 protein levels (Figures 3A,B) and CLDN2 gene expression (Figure 3D) but not for CLDN4, CLDN16, CLDN19, and CLDN23 mRNAs (Figures 3C,E; Table 1). Protein levels of CLDN4 were significantly higher in HWE under both TN and HS conditions than in the LWE line (Figures 3A,B). Heat stress increased mRNA abundances of CLDN2 and CLDN16 only in the HWE line (Figures 3D–F). Neither HS nor line affected the duodenal expression of CLDN19 or CLDN23 (Table 1).

**FIGURE 4:**
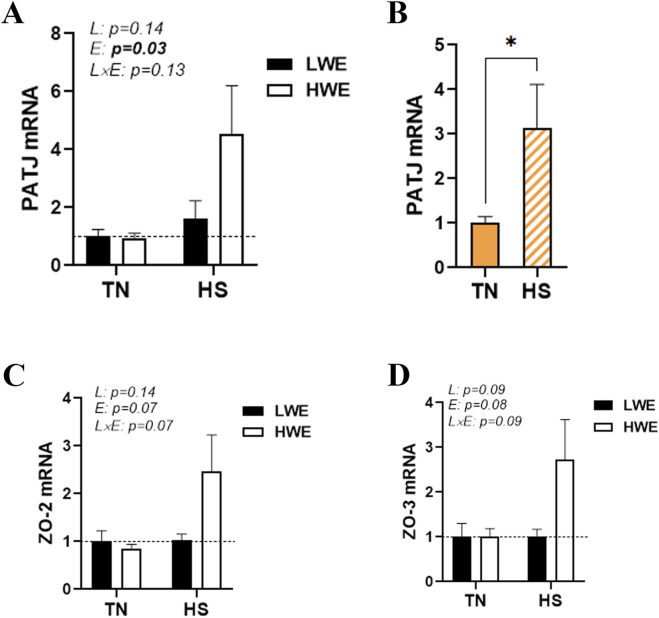
Effect of HS on the duodenal expression of PALS1-associated tight junction protein and zonula occludens in HWE and LWE lines. Duodenal expressions of PATJ **(A,B)**, ZO-2 **(C)**, and ZO-3 **(D)** were determined using qPCR and 2^−ΔΔCt^, with 18S rRNA as a housekeeping gene and LWE-TN or TN as a calibrator. Data are presented as the means ± SEM (n = 6/line/environmental condition). * indicates a significant difference at *p* < 0.05. E, environment; HS, heat stress; HWE, high-water-efficient; L, line; L × E, line-by-environment interaction; LWE, low-water-efficient; PATJ, PALS1-associated tight junction protein; TN, thermoneutral; ZO, zonula occludens. A bold *p-*value indicates a significant difference.

Heat stress also significantly upregulated the duodenal expression of PALS1-associated tight junction protein (*PATJ*), and this effect was more obvious in HWE than in the LWE line (Figures 4A,B). Although the difference was not statistically discernible, the expressions of zonula occludens *ZO-2* and *ZO-3* were also induced by HS only in the HWE line (*p* = 0.07 and *p* = 0.08 for ZO-2 and ZO-3, respectively) (Figures 4C,D). The duodenal expressions of cingulin (*CGN*), occludin (*OCLN*), and junctional adhesion molecule alpha (*JAMA*) were not affected by either HS or the bird line (Table 1).

### 3.3 Effect of HS on the duodenal expression of gap junction proteins in HWE and LWE birds

**FIGURE 5:**
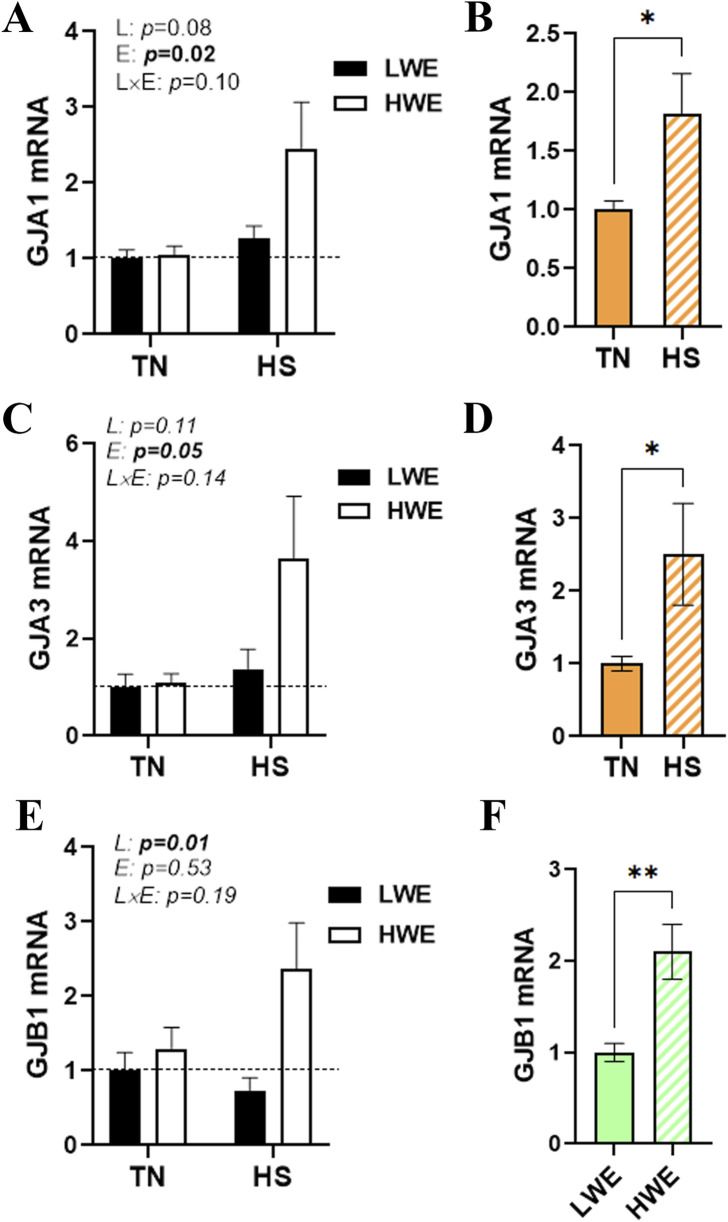
Effect of HS on the duodenal expression of gap junction proteins in HWE and LWE birds. Duodenal expressions of GJA1 **(A,B)**, GJA3 **(C,D)**, and GJB1 **(E,F)** were determined using qPCR and 2^−ΔΔCt^, with 18S rRNA as a housekeeping gene and LWE-TN, TN, or LWE as a calibrator. Data are presented as the means ± SEM (n = 6/line/environmental condition). * indicates a significant difference at *p* < 0.05. E, environment; GJA, gap junction protein alpha; GJB1, gap junction protein beta 1; HS, heat stress; HWE, high-water-efficient; L, line; L × E, line-by-environment interaction; LWE, low-water-efficient; TN, thermoneutral. A bold *p-*value indicates a significant difference.

There was no significant interaction (line x environment) effect for gap junction protein alpha (*GJA1* and *3*), gap junction beta 1 (*GJB1*), gap junction gamma 2 (*GJC2*), and gap junction delta 2 (*GJD2*) (Figures 5A,C,E; Table 1). Heat stress significantly induced duodenal mRNA abundances of *GJA1* and GJA3, and this effect was more notable in HWE than in the LWE line (Figures 5B,D). The water-efficient (HWE) broilers exhibited a significantly higher duodenal expression of GJB1 than their LWE counterparts (Figures 5E,F). The duodenal expressions of *GJC2* and *GJD2* remained unchanged between both lines under both environmental conditions (Table 1).

### 3.4 Effect of HS on the duodenal expression of adherens junctions in HWE and LWE birds

**FIGURE 6:**
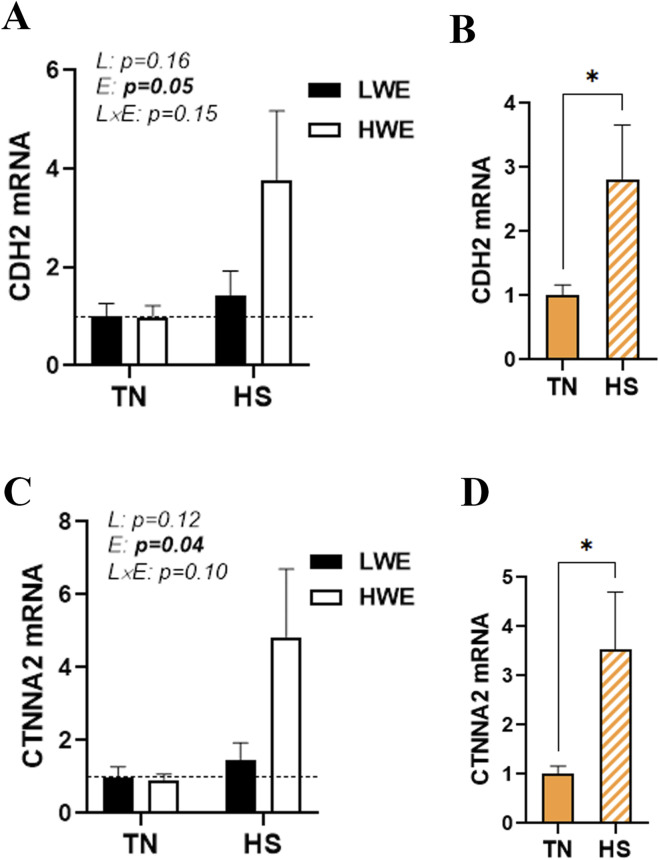
Effect of HS on the duodenal expression of adherens junction proteins in HWE and LWE birds. Duodenal expressions of CDH2 **(A,B)** and CTNNA2 **(C,D)** were determined using qPCR and 2^−ΔΔCt^, with r18S as a housekeeping gene and LWE-TN or TN as a calibrator. Data are presented as the means ± SEM (n = 6/line/environmental condition). * indicates a significant difference at *p* < 0.05. CDH2, cadherin; CTNNA2, catenin alpha 2; E, environment; HS, heat stress; HWE, high-water-efficient; L, line; L x E, line-by-environment interaction; LWE, low-water-efficient; TN, thermoneutral. A bold *p-*value indicates a significant difference.

There was no significant line-by-environment interaction effect on the duodenal expression of cadherins (CDH1/2), catenins (CTNNA2 and B1), afadin (AFDN), and nectin 1 (Figure 6; Table 1). As shown in Figures 6A–D, HS caused a significant upregulation in the duodenal expressions of *CDH2* and *CTNNA2*, and this effect was stronger in HWE than in LWE birds. The duodenal expressions of *CDH1*, *CTNNB1*, *AFDN*, and nectin 1, however, were not affected by either HS or line (Table 1).

### 3.5 Effect of HS on the duodenal expression of desmosomes in HWE and LWE birds

There was no significant line-by-environment interaction effect on the duodenal expression of desmoglein 2 and 4 (*DSG2* and *4*) (Table 1). The expressions of *DSG2* and *4* remained unchanged between the HWE and LWE lines under both environmental (TN and HS) conditions (Table 1).

## 4 Discussion

The small intestine plays a vital physiological role in digestion and nutrient absorption from ingested food. TJs hold the single layer of polarized columnar cells in the intestinal tract together and form a physical barrier in the organism. The intestinal tract is vulnerable to HS. Previous studies have shown that HS disrupts intestinal barrier integrity, leading to inflammation and leaky gut syndrome (Ncho, 2025; González-Mariscal, 2022; Brugaletta et al., 2022). In this study, morphometric analysis using H&E staining revealed a marked reduction in villus height under the HS condition, which is in agreement with the findings described by Mazzoni et al. (2022). Although no differences in crypt depth were observed in the duodenum in LWE and HWE under the HS condition, the crypt depth of HWE was slightly increased relative to that of LWE. The intestinal crypt presents stem cells, which proliferate and differentiate to maintain the self-renewal of the villus (Santos et al., 2019). This increase in crypt depth was probably attributed to a higher generation of new epithelial cells in HWE lines.

Intestinal epithelial cells interact with each other through TJs, gap junctions, adherens junctions, and desmosomes and form a functional physical barrier that maintains gut integrity and homeostasis (González-Mariscal, 2022). In this study, several tight junction genes (*CLDN1*, *CLDN5*, *CLDN8*, *CLDN16*, *PATJ*, and *CLDN22*), gap junction genes (*GJA1* and *GJA3*), and adherens junction genes (*CDH2* and *CTNNA2*), along with CLDN4 proteins, were significantly upregulated in response to HS exposure with higher amplitude in the HWE line. Although CLDN1 contributes to forming the tight junction network and regulating paracellular permeability, its upregulation is often associated with both increased intestinal permeability and enhanced integrity, depending on the (patho)physiological context (Wang et al., 2012; Pope et al., 2014; Iraha et al., 2013). Moreover, CLDN1’s role in inflammation is complex and can vary depending on the context and location. For instance, increased expression of CLDN1 was observed in ulcerative colitis and Crohn’s disease and has been found to be associated with inflammation (Poritz et al., 2011). However, other studies reported that increasing CLDN1 expression might improve barrier function and decrease inflammation in atopic dermatitis (Be et al., 2020). Similarly, in poultry, although the exact function of CLDN1 needs to be defined, studies reported inconsistent (upregulation, downregulation, or no effect) results regarding HS effects (Greene et al., 2025; Thi Dung et al., 2023; Del Ve et al., 2020). In contrast to the duodenum here, we have shown in a previous study that HS did not affect CLDN1 expression in the ileum, which suggests a tissue-specific regulation of CLDN1, adding another layer of complexity to its function. Interestingly, CLDN1 has been found to play a role in trans-epithelial water retention and loss (Barmeyer et al., 2017). It is, therefore, reasonable to speculate that the upregulation of CLDN1 might enhance duodenal barrier integrity and lower duodenal trans-epithelial water loss in HWE birds that exhibited lower serum fluorescein isothiocyanate (FITC) levels and overall better gut integrity under HS conditions (Greene et al., 2025).

Claudin 5 has been predominantly studied in the context of the blood–brain barrier (BBB) as a gatekeeper of neurological function, but an increasing number of studies have demonstrated its important role in intestinal barrier integrity. It has been shown that the upregulation of CLDN5 prevents inflammation and protects intestinal epithelial cells from tumorigenesis (Zhang et al., 2022). Watari et al. (2016) showed that increased expression of CLDN5 by checkpoint kinase 1 (Chk1) activation enhances intestinal epithelial function in Caco-2 cells. Additionally, it has been shown in *Campylobacter jejuni*-infected IL10 knockout mice and in an *in vitro* model that resveratrol reduced leaky gut syndrome by upregulating CLDN5 expression (Lobo de Sa et al., 2021). Barmeyer et al. (2017) showed that epithelial barrier dysfunction and leaky gut in lymphocytic colitis occur through downregulation and redistribution of CLDN5.

Claudin 8 mRNA and proteins were found to be moderately expressed in the ileum and colon but were absent in the jejunum and duodenum in rodents and humans (Lameris et al., 2013; Holmes et al., 2006). In this study, we showed that the *CLDN8* gene is expressed in the chicken duodenum, which indicates a species-specific expression, localization, or function. It has been shown that pro-inflammatory cytokines, such as TNF-α and IL-15, alter CLDN8 expression and distribution, resulting in lymphocytic colitis, which is characterized by epithelial barrier impairment, leak-flux diarrhea, and abnormal fluid absorption (Barmeyer et al., 2017). Furthermore, it has been shown that CLDN8 expression was downregulated and redistributed off the tight junction in patients with active Crohn’s disease that is typified by impaired intestinal barrier function (Zeissig et al., 2007). Marincola Smith et al. (2021) further demonstrated a decreased expression of CLDN8 in inflammatory bowel disease, which is denoted by a defective barrier function. Although CLDN22 contributes to the general function of claudins in regulating intestinal paracellular permeability and barrier integrity (Wang et al., 2016), existing published research and scholarly materials are scarce, and its specific functions are still unknown.

Heat stress also induced the expression of pore-forming proteins, including CLDN2, CLDN4, and CLDN16, mainly in the HWE line. There appears to be conflicting evidence regarding the precise roles of these proteins in gut integrity, which are complex and context-dependent (Luettig et al., 2015; Liu et al., 2013; Ahmad et al., 2023; von Buchholz et al., 2021). For instance, Oami et al. (2024) reported that CLDN2 upregulation induces intestinal permeability and dysbiosis in sepsis. Ahmad et al. (2014), however, showed that increasing CLDN2 expression confers resistance to epithelial injury and protects mice from colitis. Similarly, CLDN4 plays a complex and often paradoxical role in intestinal barrier integrity, acting as a component of TJs that maintain the barrier integrity but also being upregulated in some gastrointestinal diseases, which can induce intestinal permeability (Okamoto et al., 2023; Boschetti et al., 2019; Assimakopoulos et al., 2006). CLDN16, also known as paracellin-1, is primarily known for its role in kidney magnesium and calcium reabsorption (Hou et al., 2009; Negri, 2015; Deluque et al., 2025; Hou et al., 2007); however, emerging studies suggest that it may play a role in the gut. Ozden et al. (2010) reported a co-localization of CLDN16 and goblet cells in developing chick embryo intestine, indicating a potential role in mucus secretion and/or membrane remodeling. As CLDN2, 4, and 16 form paracellular channels (Wilmes et al., 2014), it is possible that they enhance, as in the kidney, water reabsorption in the intestine and thereby ameliorate water homeostasis in HWE birds. Of particular interest, duodenal CLDN4 protein levels, but not mRNA, were higher in HWE than in the LWE line under both environmental conditions, and they were induced by HS only in HWE birds. This suggests that, although further in-depth investigations are needed, CLDN4 might play a role in cellular stress response, such as hypertonic, ER, and/or oxidative stresses (Lanaspa et al., 2008; Pao et al., 2021; Campos-Blazquez et al., 2023), which seem to be enhanced in the HWE line. The discordance between CLDN4 mRNA and protein levels is a known phenomenon and is, therefore, not surprising. This discordance could be driven by complex post-transcriptional, post-translational, and epigenetic regulatory mechanisms. For instance, CLDN4 is post-transcriptionally regulated by long non-coding RNAs, leading to increased protein levels even without a significant change in its mRNA expression (Song et al., 2017). Furthermore, CLDN4’s stability can be affected by interaction with other proteins, including CLDN2, 3, and 8 (Van Itallie and Anderson, 2013), or by proteolytic cleavage (Gong et al., 2014).

Heat stress induced the duodenal expression of PATJ, particularly in HWE birds. The crumbs/PALS1/PATJ complex plays a critical role in maintaining intestinal epithelial cell polarity (Penalva and Mirouse, 2012), and PATJ binds to other tight junction proteins, such as claudins, to stabilize tight junction formation (Michel et al., 2005). Blocking PATJ induces an increase in the transepithelial flux of mannitol from the basolateral to the apical compartment in the human intestinal epithelial cell line (Lunardi et al., 2009). Together, this result indicates that the increased expression of PATJ might enhance tight junction formation and stability in heat-stressed HWE birds, resulting in better gut integrity (Greene et al., 2025).

In addition to tight junctions, intestinal homeostasis is also dictated by intercellular communication, which is typically governed by gap junctions composed of two hemichannels of neighboring cells that control the diffusion of small and hydrophilic chemical substances between adjacent cells (Alexander and Goldberg, 2003). The gap junction is composed of two systems: connexins with more than 21 different protein variants and pannexin with 3 types that have been identified over the years (Panchin et al., 2000; Diezmos et al., 2016). In this study, our data showed that HS induced the expression of the *GJA1/3* and *GJB1* genes, which encode connexins 43, 46, and 32, respectively, and this induction was more pronounced in HWE, similar to the abovementioned tight junction proteins. Although their roles in the chicken gut remain unknown, connexins 32, 43, and 46 are believed to play key roles not only in maintaining epithelial barrier function by affecting tight junction protein production (Nagasawa et al., 2006; Johnson et al., 2018; Murata et al., 2005) but also in regulating intestinal nerve transmission, motility, transit, and pacing (Doring et al., 2007; McClain et al., 2014). It is worth noting that in contrast to the duodenum segment, the expression of the ileal *GJA1* gene was not affected by HS, but it was upregulated in HWE birds (Greene et al., 2025), suggesting tissue/cell-specific regulation of annexins (Maes et al., 2015).

AJ plays a crucial role in initiating and maintaining intercellular adhesion, orchestrating the organization of the actin cytoskeleton beneath the membrane, and serving as a signaling hub for cell transduction and regulation of gene transcription (Garcia et al., 2018). The adherens junction is primarily composed of cadherins (CDH1 and CDH2), β-catenin, and α-catenin. In our experimental condition, HS upregulated the duodenal expressions of *CDH2* and *CTNNA2* only in the HWE line. Cadherins are pivotal for cell-to-cell adhesion and, thereby, play a vital role in maintaining the structural integrity of the intestinal epithelial layer and, consequently, intestinal barrier integrity (Schneider et al., 2010; Baumgartner, 2013). Through Wnt signaling, catenins regulate intestinal stem cell renewal and proliferation, repair and regeneration of injured gut tissue, prevent inflammation and mucosal damage, and maintain intestinal barrier integrity (Smalley-Freed et al., 2010; Fevr et al., 2007; Mehta et al., 2015; Eum et al., 2023). It is also worth mentioning that CTNNA2 was induced by HS in a similar way in the ileum; however, CDH2 was not affected, suggesting again a tissue-specific regulation.

In conclusion, this is the first report, to our knowledge, showing that chronic HS modulates the duodenal expression of tight junction, gap junction, and adherens junction proteins in an environmental condition- and/or line-dependent manner. The upregulation of claudins (*CLDN1*, *2*, 4, *5*, *8*, *16*, and *22*), *PATJ*, *GJA1/3*, *GJB1*, *CDH2*, and *CTNNA2* in HS suggests an improvement in gut integrity in HWE birds, which was evidenced by lower serum FITC levels (Greene et al., 2025), better water efficiency, and better growth performances (Aloui et al., 2024). It is important to mention here that we measured only mRNA and acknowledge the limitation of not measuring protein levels due to the lack of specific antibodies that cross-react with chicken proteins. Proteins are the workhorses of the cells, and it is probable that their regulation by HS and/or the chicken line is not coordinated with the mRNA levels, as is the case for CLDN4.

## Data Availability

The original contributions presented in the study are included in the article/supplementary material; further inquiries can be directed to the corresponding author.
